# Gelatine nanostructured lipid carrier encapsulated FGF15 inhibits autophagy and improves recovery in spinal cord injury

**DOI:** 10.1038/s41420-020-00367-y

**Published:** 2020-12-02

**Authors:** Yibo Ying, Guangheng Xiang, Min Chen, Jiahui Ye, Qiuji Wu, Haicheng Dou, Sunren Sheng, Sipin Zhu

**Affiliations:** 1 Department of Orthopaedics, The Second Affiliated Hospital and Yuying Children’s Hospital of Wenzhou Medical University, Wenzhou, Zhejiang 325000 China; 2 The Second School of Medicine, Wenzhou Medical University, Wenzhou, 325027 China

**Keywords:** Neuroscience, Physiology

## Abstract

Gelatine nanostructured lipid carriers (GNLs) have attracted increasing attention due to their biodegradable status and capacity to capture various biologically active compounds. Many studies demonstrated that fibroblast growth factor therapies after spinal cord injury (SCI) can be used in the future for the recovery of neurons. In this study, the therapeutic effects of GNL-encapsulated fibroblast growth factor 15 (FGF15) and FGF15 were compared in SCI. The FGF15-GNLs had 88.17 ± 1.22% encapsulation efficiency and 4.82 ± 0.12% loading capacity. The effects of FGF15-GNLs and FGF15 were assessed based on the Basso–Beattie–Bresnahan (BBB) locomotion scale, inclined plane test and footprint analysis. Immunofluorescent staining was used to identify the expression of autophagy-associated proteins, GFAP (glial fibrillary acidic protein) and neurofilament 200 (NF200). FGF15-GNLs use enhanced the repair after SCI compared to the effect of FGF15. The suppression of autophagy-associated proteins LC3-II and beclin-1, and p62 enhancement by FGF15-GNLs treatment were more pronounced. Thus, the effects of FGF15-GNLs on the recovery after SCI are related to the inhibition of autophagy and glial scar, and promotion of nerve regeneration in SCI.

## Introduction

Spinal cord injury (SCI) and overwhelming complications after SCI are very damaging and provoke serious sensory, motor and neurological dysfunction below the injured segment. SCI has become a global problem^1^. The damage of traumatic SCI to the body is not limited to the direct mechanical impact on the spine and is associated with numerous life-threatening complications in the spinal cord, which are caused by a complex chain of cascade amplification reactions leading to progressive cell death and spinal cord damage. Associated disorders include ionic homoeostasis, local oedema, ischaemia, focal haemorrhage, free radical stress and inflammatory response^2–4^. Currently, many neuroprotective therapies, including surgery, methylprednisolone and blood pressure augmentation, aim to decompress and stabilize the injury, prevent secondary complications and management of existing symptoms^3–5^. Previous studies have indicated that autophagy plays a crucial role in the secondary injury in animal models and human tissue, gradually resulting in the spinal cord regeneration. Thus, autophagy may play an important role in the therapeutic strategies aimed to improve the recovery of patients with SCI^6–8^.

Autophagy is the process in which cells coat their damaged organelles or proteins in a double membrane structure and transport them to the lysosomes for degradation^9^. Autophagy is a dynamic biological process that is very important in the growth, development and differentiation of the cells^10–12^. However, excessive autophagy results in the accumulation of abnormal proteins in the cells that block the normal activities of the cells. Autophagy participates in the regulation of many diseases including SCI. Autophagy is induced by a variety of stress stimuli, including endoplasmic reticulum stress, hypoxia, DNA damage, redox stress and mitochondrial damage^13,14^. Many therapeutic interventions in SCI use neurotrophic factors and are mainly focused on reducing the damage caused by excessive autophagy in the spinal cord cells. Events that result in secondary injury, such as exposure to free radicals, accumulation of unfolded or misfolded proteins and hypoglycaemia, are boosted by autophagy. Excessive autophagy hinders the stable growth and effective repair of nerve cells after SCI^15^. Sustained or massive autophagy contributes to death of autophagic cells. During SCI, prolonged autophagy within adverse local microenvironment eventually results in neuronal death. Thus, disruption of autophagy can help the cells to deal with this situation. Autophagy is involved in cell death, especially in the neurons and spinal cord glia, after SCI^16,17^.

Two classical pathways to regulate autophagy include the mammalian target of rapamycin (RAPA) pathway regulated by PI3K-AKT and PTEN phosphatase and the beclin-1/Vps-34/Vps-15 complex pathway^18^. Previous studies demonstrated that overexpression of beclin-1 and microtubule-related protein light chain 3 (LC3-II) results in extreme stimulation of autophagy and induces cell death. LC3-II is located on the autophagic vesicles in mammalian cells and is used as a marker to specifically track autophagy^19^. Stimulation-induced p62 is a cellular protein involved in autophagy; p62 is responsible for sorting of protein domains, can bind to and degrade polyubiquitin, and plays an important role in signal transduction^20^.

FGF15 is an intercellular signal molecule that plays a significant role in embryogenesis and differentiation. FGF15 is considered a promoter in the formation of lesions. Some studies demonstrated that FGF15 is a positive factor in cell repair. FGF15 is the key member of the neurotrophin family and is an important regulator of neuronal survival, development, function and plasticity^21,22^. Neuroprotective effects of FGF15 were reported to improve the recovery of SCI. However, the application of FGF15 is frequently obstructed in the clinic, because FGF15 is unstable and vulnerable to temperature, pH and other environmental, physical and chemical factors^23,24^. Limited shelf life and high production cost of FGF15 make it difficult to be widely used. Liposomes consist of hydrated lipid bilayer vesicles generally characterized by low toxicity and biodegradability. Liposomes can be used as a successful drug delivery system for FGF15 in the treatment of SCI^25^.

In this study, we prepared and characterized FGF15-GNLs to stabilize FGF15 and develop a sustained-release transdermal drug delivery system of FGF15. The studies of FGF15-GNLs in SCI have not been reported. We also investigated whether the protective effect of gelatine nanostructured lipid carriers (GNLs) encapsulating FGF15 is related to inhibition of autophagy. Our results suggest that FGF15-GNLs can have therapeutic potential in the treatment of SCI.

## Materials and methods

### Reagents and antibodies

Dulbecco’s modified Eagle’s medium (DMEM) was from Invitrogen (Carlsbad, CA, USA). Antibodies (LC3-II (ab192890), p62 (ab109012), beclin-1 (ab207612), glial fibrillary acidic protein (GFAP) (ab7260) and NF200 (ab82259)) were from Abcam (Cambridge, UK). Goat anti-rabbit (ab150077) and anti-mouse (ab150117) IgG-488, and goat anti-rabbit IgG-HRP (ab7090) were from Abcam (Cambridge, UK). An enhanced chemiluminescence kit was from Bio-Rad (Hercules, CA, USA).

### Preparation of GNLs

GNLs loaded with FGF15 were prepared by water-in-water emulsion and freeze-drying technology. The process is described in Supplemental Experimental Procedures. The final FGF15 concentration in FGF15-GNLs suspensions was 2 mg/mL.

### Characteristics of GNLs

The encapsulation efficiency, loading capacity and bioactivity of FGF15 and GNLs were assayed as described in detail in Supplemental Experimental Procedures.

To identify the encapsulation efficiency of FGF15 liposomes, the supernatant from ~1.5 ml of the FGF15-GNLs dispersion was collected after centrifugation at 10,000 × *g* for 40 min and diluted for determination of FGF15 content. The drug encapsulation efficiency was calculated as follows:

Encapsulation efficiency (%) = (total amount of FGF15 − amount of FGF15 in supernatant)/total amount of FGF15 added × 100%.

### Animal model of SCI and FGF15-GNLs administration

Young adult female Sprague–Dawley rats ~8 weeks of age (weighing 220–250 g) were provided by the Animal Center of Chinese Academy of Sciences, Shanghai, China. Animals were housed at 23–25 °C with free access to water and food for 7 days before the experiment. The protocol of the animal use and care was approved by the Animal Care and Use Committee of Wenzhou Medical College and was strictly compliant with the Guide for the Care and Use of Laboratory Animals of the National Institutes of Health. Anaesthesia was administered to all rats by injection of 10% chloral hydrate (3.5 ml/kg). In ~10 min, the animals were placed on a cork platform. The skin was incised along the posterior median line and eight to ten thoracic vertebrae were exposed. Acute SCI was modelled by striking the T9 segments of the spinal cord with a hammer (10 g) allowed to freely fall from 25 mm height. The same procedures were applied to the sham group rats without hitting the spinal cord. Animal care and handling involved massage of the bladder to induce urination twice daily until the reflex bladder function was rebuilt by using cefazolin sodium (50 mg/kg, intraperitoneally (i.p.)).

FGF15 was from Sigma-Aldrich (St. Louis, MO, USA). GNLs were developed in-house in Wenzhou Medical University of Medicine. After SCI, the stock solution was diluted with 0.9% NaCl and administered intravenously at a dose of 20 µg/kg/day once daily until the animals were killed. Control group of animals received the same volume of NaCl at the corresponding times. Uniform treatment with vehicle or FGF15-GNLs was performed until the data were finally analysed. Each experimental animal was subjected to daily nursing procedures involving passive activity of the hind legs twice per day.

### Cell culture and treatment

NSCs were from the American Type Culture Collection and were cultured in the neural stem cell culture medium (Chiscientific, Jiangsu, China). Neural stem cell medium (100 ml) included 96 ml DMEM, 2 ml B27, 1 ml glutamine, 1 ml horse serum, 2 mM glutamine, penicillin/streptomycin, 100 µl heparin, 20 µl basic fibroblast growth factor, and 10 µl epidermal growth factor. Cells were incubated in a humidified incubator at 5% CO_2_ and 37 °C. The impact of RAPA (100 nM; Cell Signaling Technology, USA) on the survival and proliferation of neural stem cells was determined. Based on our previous experience, RAPA (100 nM; Cell Signaling Technology, USA) was added the cells to test the impact of autophagy on neural stem cells at 12 and 24 h. All experiments were performed in triplicate. All cells were randomly divided into four groups, including NSCs, NSCs + RAPA, NSCs + RAPA + FGF15 and NSCs + RAPA + FGF15-GNLs.

### Behavioural recovery evaluation

To examine the recovery of the physical status after the injury, locomotion recovery assessment was performed by personnel who were blinded to the animal conditions. The Basso–Beattie–Bresnahan (BBB) locomotion scale was used to describe and evaluate hindlimb movement function; BBB is a 22-point scale (scores 0–21) that logically tacks the recovery of hindlimb function ranging from 0 points, representing no observed movements of hindlimbs, to 21 points, indicating normal rodent ambulation.

The inclined plane was used with a 6 mm-thick rubber pad placed on the surface of the inclined plate. Rats were placed according to the direction vertical to the body axis and the longitudinal axis of the inclined plate. The angle between the swash plate and the horizontal plane was gradually increased until the rat stayed on the plate for 5 s, and the inclination angle was recorded.

In the footprint test, the front and hind paws were immersed in red and blue dyes, respectively. Rats were placed on a 7.5 cm × 100 cm runway with white paper and were allowed to pass from one end of the runway to the other. The footprints were scanned and analysed to assess the motor ability.

Video recording of locomotor function. Each group of rats were photographed using a camera (Leica), while walking through a 1 m-long glass runway with markers on the hindlimbs to estimate hip, knee, ankle and foot positions. The following parameters were used to evaluate locomotion: (1) weight support (height; hip height minus trunk width, equal to the torso gap on the ground), (2) leg-extensor spasms (quantified as the time the foot is overstretched and dragged relative to the foot cycle), (3) the number of footsteps (the number of footsteps calculated relative to the number of forefoot steps) and (4) the posture of the foot (measurement of the foot offset behind the hip at the tip of the ankle). The walking step rhythm was defined by the front leg (front leg steps/second).

### Haematoxylin–eosin staining, immunohistochemistry and histology

Deep anaesthesia was performed in the sham and SCI groups (*n* = 6) with 10% chloral hydrate (3.5 ml/kg, i.p.); animals were perfused with 0.9% NaCl followed by perfusion with 4% paraformaldehyde in 0.01 M phosphate-buffered saline (PBS, pH 7.4) at 60 days. The T7–T9 spinal cord lesions around the epicentre were stripped and incubated in cold 4% paraformaldehyde overnight; then, the samples were embedded in paraffin. Transverse paraffin sections (thickness 5 μm) were used for histopathological examination by haematoxylin and eosin (H&E) staining. For Nissl staining, the sections were incubated in 1% cresyl violet. Two stained sections were inspected and scanned using a light microscope.

### Immunofluorescence staining

The frozen sections were dried and baked in an incubator at a constant temperature for 4 h. Xylene was used to deparaffinize the sections twice for 10 min. After rehydration with gradient alcohol for 5 min, the sections were rinsed twice with PBS for 5 min and incubated in H_2_O_2_ for 15 min; then, the sections were washed three times with PBS for 5 min. PBS was removed and sodium citrate buffer solution (pH 6) was added; the samples were heated to 95 °C in a pressure cooker until the air was vented for 2 min. Samples were washed three times with PBS containing 0.05% Tween 20 (PBST) for 5 min at room temperature and permeabilized with 0.5% Triton X-100 for 20 min; sections were rinsed three times with PBST for 5 min. Slides were blocked with 1% bovine serum albumin (1% 0.01 M phosphate-buffered saline (PBS) dilution) for 30 min at room temperature and incubated with suitable primary antibodies overnight at 4 °C in the same buffer. The nuclei were stained with 4′,6-diamidino-2-phenylindole (0.25 μg/ml). Primary antibodies against various targets were used: anti-LC3-II (1 : 200; Abcam, UK), anti-p62 (1 : 300; Abcam, UK), anti-beclin-1 (1 : 500; Abcam, UK), anti-NF200 (1 : 1000, Abcam, UK) and anti-GFAP (1 : 500, Abcam, UK). After incubation with a primary antibody, the sections were washed with PBST three times for 5 min at room temperature; Alexa Fluor 488 goat anti-mouse/rabbit or Alexa Fluor HRP goat anti-rabbit secondary antibodies (1 : 500; Abcam, UK) were incubated in the dark for 1 h at 37 °C. The samples were washed three times with PBST for 5 min in the dark. The fluorescent quencher was added and the slides were sealed and stored at 4 °C. The samples were imaged using a Nikon ECLIPSE Ti microscope (Nikon, Tokyo, Japan).

### Statistical analysis

The differences between the treatment and control groups were determined based on the mean ± SEM. Statistical significance was estimated by *t*-test in two experimental groups. In two other groups, repeated analysis of variance was used for evaluation of the data followed by Dunnett’s post hoc test. The post hoc analysis included Bonferroni multiple comparison test. Statistical significance was estimated based on *P*-value < 0.05.

## Results

### Physicochemical bioactivity of GNLs and the particles loaded with FGF15

Table 1 shows the characteristics of GNLs loaded with or without FGF15. The dynamic light scattering indicated that the average particle size of GNLs and FGF15-GNLs was 122.23 ± 1.28 and 181.57 ± 1.31 nm, respectively. The polydispersity index (PDI) indicates the particle size distribution; lower PDI values were observed in GNLs and FGF15-GNLs, indicating that GNLs and FGF15-GNLs were close to a monodisperse stable systems.

**Table 1:** Characterization of GNLs loaded with or without FGF15 (*n* = 6).

Formulation (nm)	Particle size	PDI	Zeta potential (mV)	Encapsulation efficiency (%)	Loading capacity (%)	Bioactivity (×10^5^ IU/ml)
GNLs	122.23 ± 1.28	0.28 ± 0.05	−18.15 ± 1.90	—	—	—
FGF15-GNLs	181.57 ± 1.31*	0.10 ± 0.03*	−29.27 ± 1.31*	88.17 ± 1.22	4.82 ± 0.12	5.79 ± 0.36

*PDI* Polydispersity Index.

*p < 0.05 (FGF15-GNLs vs. GNLs).

After FGF15 loading, mean nanoparticle size was increased but remained below 200 nm (Table 1). Zeta potential is an important indicator of the physical stability of nanoparticles. Nanoparticles with high absolute value of the zeta potential are electrically stable, whereas nanoparticles with a low absolute value of the zeta potential tend to fluctuate. Table 1 shows that FGF15-GNLs had a stronger negative charge on the surface compared with that of GNLs, which had potential values below −25 mV, indicating that the FGF15-GNLs dispersion is more stable. The encapsulation efficiency and loading ability of FGF15-GNLs were 88.17 ± 1.22% and 4.82 ± 0.12%, respectively (Table 1). The bioactivity of FGF15-GNLs was (5.79 ± 0.36) × 10^5^ IU/ml, which indicated that the preparations of FGF15-GNLs have better physiological properties.

### FGF15-GNLs increase the migration and repair of NSCs

**Fig. 1:**
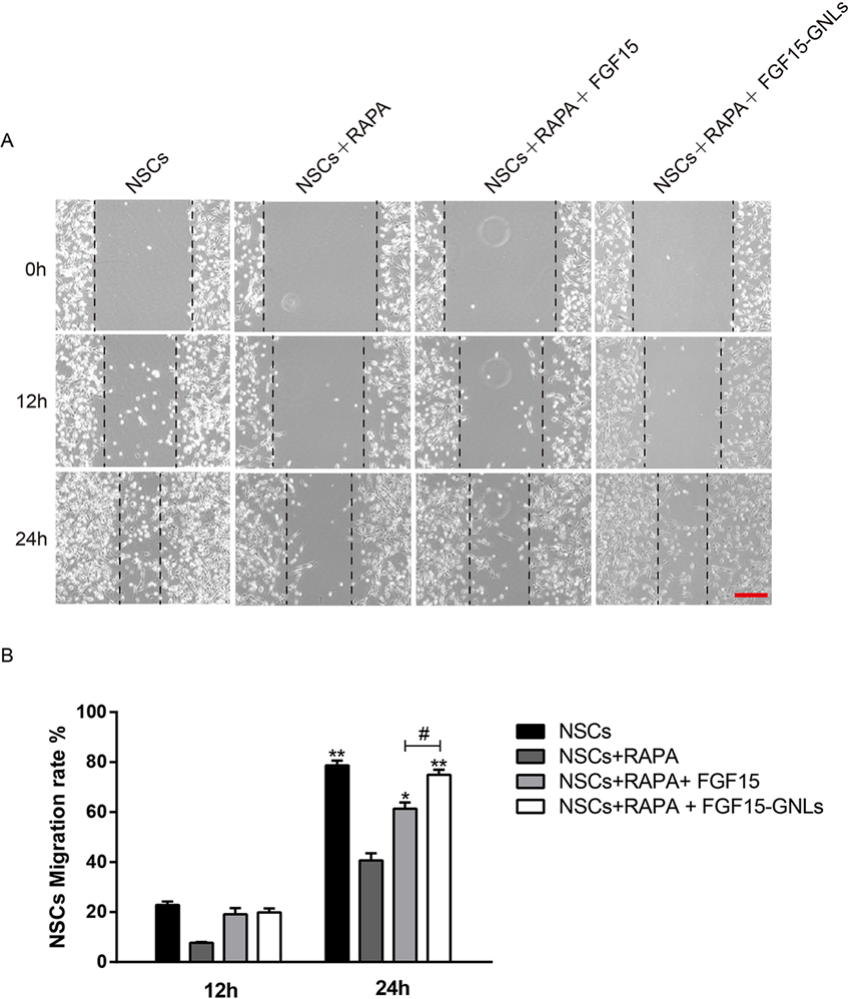
FGF15-GNLs inhibits autophagy-induced autophagic cell death in SCI model. **A** Images of wound-healing of NSCs group, NSCs + RAPA group, NSCs + RAPA + FGF15 group and NSCs + RAPA + FGF15-GNLs group. These images were captured by an inverted phase-contrast microscope at 0, 12 and 24 h after the scratch, scale bar = 100 μm. **B** The NSCs migration ratio of the NSCs group, NSCs + RAPA group, NSCs + RAPA + FGF15 group and NSCs + RAPA + FGF15-GNLs group.

Wound-healing assay results after 12 and 24 h scratch in the NSCs, NSCs + RAPA, NSCs + RAPA + FGF15 and NSCs + RAPA + FGF15-GNLs groups are shown in Fig. 1A. The NSCs + RAPA group had larger cell-free area compared with that of the NSCs group; treatment with NSCs + RAPA + FGF15-GNLs reduced cell-free area and produced faster migration compared to those in the NSCs + RAPA group. Although the NSCs + RAPA + FGF15 group had enhanced cell migration, the NSCs + RAPA + FGF15-GNLs group had even better characteristics (Fig. 1).

### FGF15-GNLs improves the recovery after SCI

**Fig. 2:**
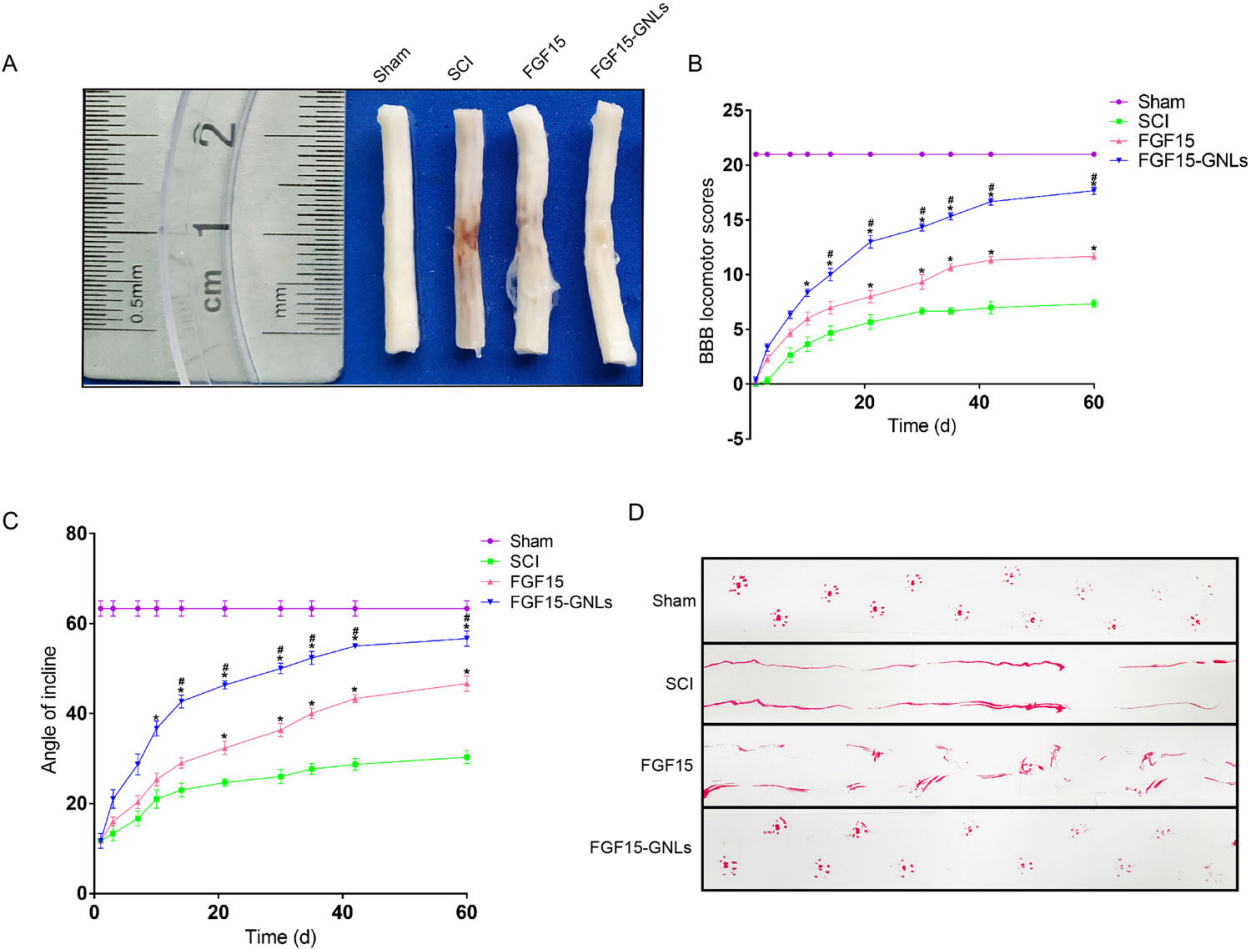
FGF15-GNLs improves the recovery of SCI. **A** Spinal cord pictorial diagram. **B** The BBB scores of sham, SCI group, FGF15 group and FGF15-GNLs group. The sham group scoring 21, means normal locomotion. *P < 0.05 vs. the SCI group, #*P* < 0.05 vs. the FGF15 group. Data are the mean values ± SEM, *n* = 6. **C** The inclined plane test scores of sham, SCI group, FGF15 group, FGF15-GNLs group. *P < 0.05 vs. the sham group or SCI group, ^#^*P* < 0.05 vs. the FGF15 group. Data are the mean values ± SEM, *n* = 6. **D** Footprint analyses of sham, SCI group, FGF15 group and FGF15-GNLs group. Sham group represents the normal movement. SCI group on behalf of the hind limbs dyskinesia after spinal cord injury.

**Fig. 3:**
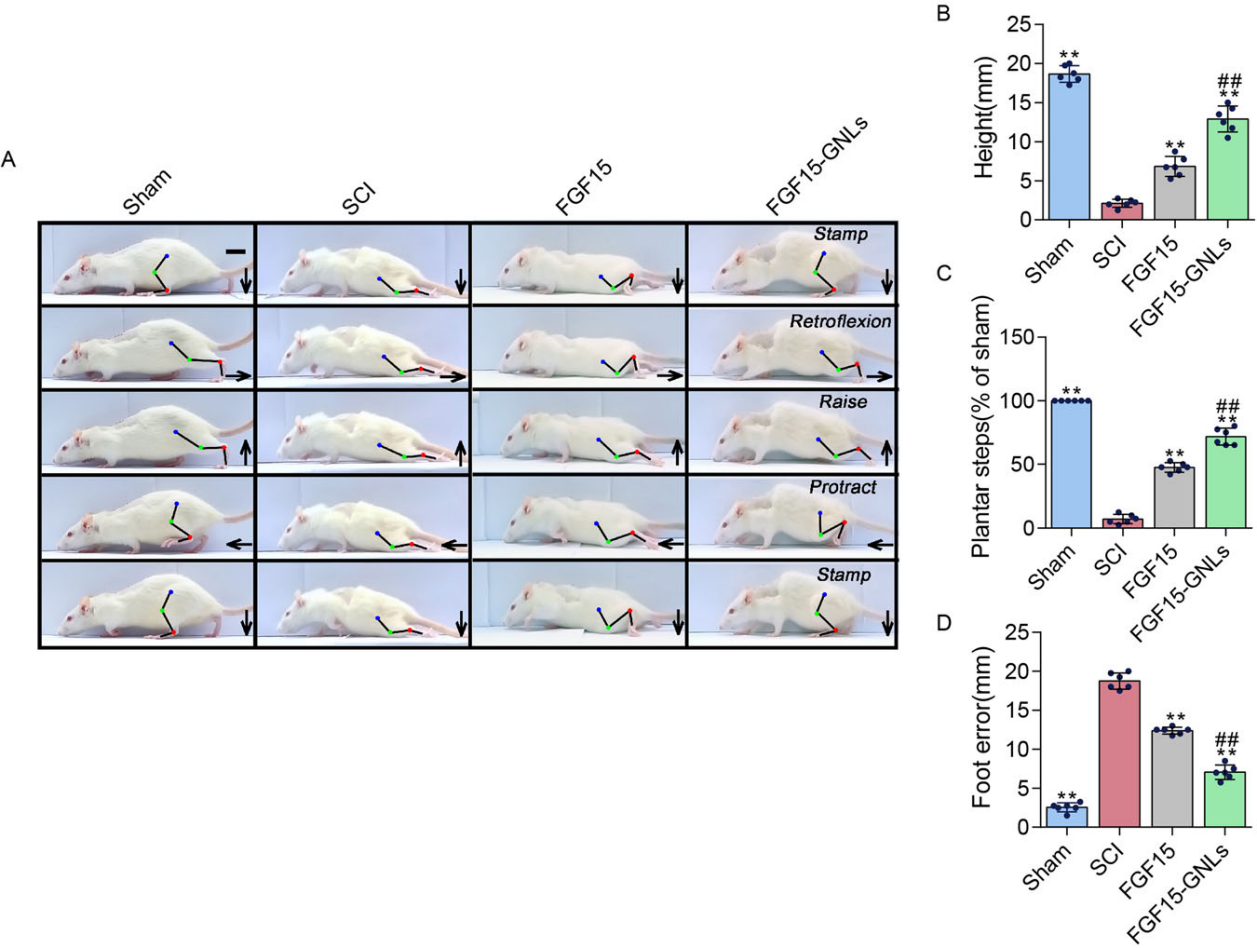
FGF15-GNLs increases locomotor function after SCI. **A** Video sequence of a rat walked 2 months after SCI. Impaired hindlimb function, while walking is evident from poor weight support (quantified as torso height above ground), leg-extensor spasms (quantified as spasm time relative to step-cycle duration), slow steps (number of hindlimb plantar steps per step cycle of the front leg) and poor foot placement (caudal to behind hip). Hip (iliac crest), knee and ankle joints are shown with dots and lines. Arrow shows foot movement. Scale bar = 20 mm. **B**–**D** The main cause of impaired hindlimb function during walking is poor weight support (quantified as the height of the trunk from the ground). Slow pace (steps of the hind legs of each hind leg cycle of the front legs). Poor foot placement (from the tail to the back of the hip). **P < 0.01 vs. the sham group or SCI group, ^##^*P* < 0.01 vs. the FGF15 group. Data are the mean values ± SEM, *n* = 6.

To evaluate the therapeutic effect of FGF15-GNLs, SCI model rats were treated with FGF15-GNLs and FGF15 by intravenous tail injection. Morphological changes in the spinal cord tissue after SCI are shown in Fig. 2A. The BBB rating scale, inclined plane test and footprint recordings, were used to test functional recovery. The BBB motor scores of FGF15, FGF15-GNLs and SCI groups were assessed at 1, 3, 7, 10, 14, 21, 28, 35, 42 and 60 days after SCI. After SCI in rats, hindlimb paralysis recovered after the growth of crus. According to the BBB scores, FGF15-GNLs significantly increased the hindlimb locomotor function. FGF15-GNLs also improved the BBB locomotor function compared to that in the FGF15 group at 1, 3, 7, 10, 14, 21, 28, 35, 42 and 60 days (*F* = 4.251) (Fig. 2B). The FGF15-GNLs treatment group had a significantly higher inclination angle determined 60 days after the injury compared with that in the FGF15 and vehicle control groups (*F* = 4.432) (Fig. 2C). Footprint analyses of the FGF15-GNLs-treated rats at 60 days after SCI showed that hindlimbs were fairly consistent and there was a little toe drag. In contrast, footprints obtained from the vehicle-treated animals showed inconsistent coordination and extensive toe dragging estimated by the ink streaks extending from both hindlimbs (Fig. 2D). Our animal model of thoracic SCI was established by injury of the T9 segments of the spinal cord to assess the locomotion recovery after SCI. Sixty days after the injury, the rats improved certain capacity of voluntary hindlimb locomotion despite the support of damaged weight, poor placement of plantar foot and slow timing of the steps partly due to leg-extensor spasms (Fig. 3). The rats treated with FGF15-GNLs had lower foot error index, higher height index and plantar steps (Fig. 3B–D).

### FGF15-GNLs increases the survival of neurons after SCI

**Fig. 4:**
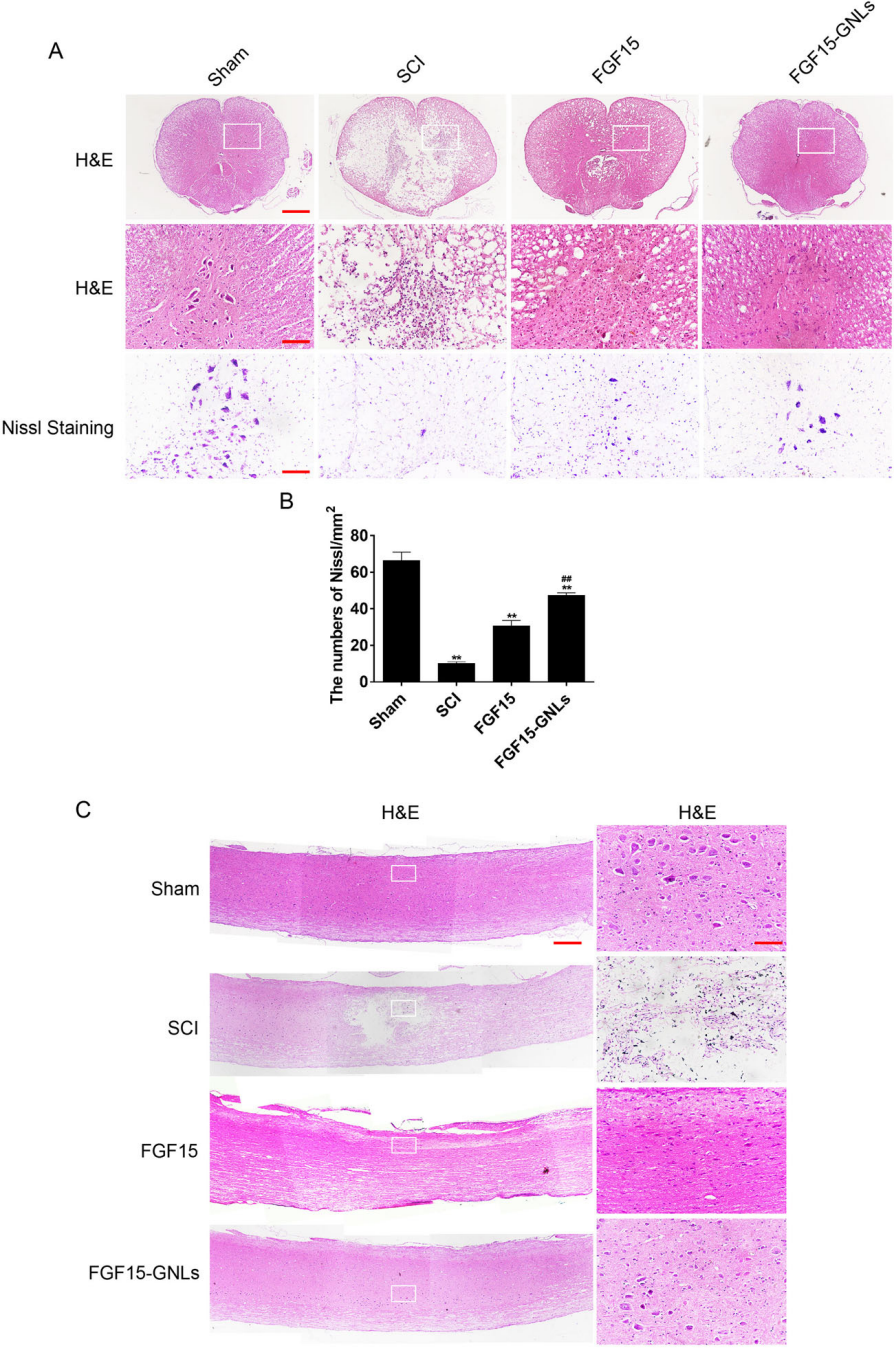
FGF15-GNLs improves the recovery of SCI. **A** H&E staining (cross-section) results for the sham group, SCI group, FGF15 group and FGF15- GNLs group, scale bar = 500 μm. A boxed region illustrates a representative region with high power images, scale bar = 100 μm. Nissl staining of the different groups, scale bar = 100 μm. **B** Analysis of the Nissl staining expressions. **P < 0.01 vs. sham group or SCI group. ^##^*P* < 0.01 vs. the FGF15 group. Data are the mean values ± SEM, *n* = 6. **C** H&E staining (longitudinal section) results for the sham group, SCI group, FGF15 group and FGF15-GNLs group, scale bar = 500 μm. A boxed region illustrates a representative region with high power images, scale bar = 100 μm.

H&E staining results of the spinal cord specimens in the sham, SCI, FGF15 and FGF15-GNLs groups 60 days after the injury are shown in Fig. 4A, C. The white and central grey tissues after SCI were gradually destroyed in the experimental groups. Treatment with FGF15-GNLs resulted in protection manifested as a reduction in necrosis, karyopyknosis and infiltration of polymorphonuclear leukocytes and macrophages compared with the patterns observed in the untreated SCI group.

The results obtained using spinal cord sections indicate that FGF15 group had some improvement; however, the FGF15-GNLs treatment was characterized by better recovery. These results emphasize the neuroprotective efficacy of FGF15-GNLs in motor neuron recovery in the spinal cord in the SCI rat model.

In contrast with the SCI group, the spinal cords of the sham group had an increase in the anterior horn, a decrease in the number of large and moderate neurons and a decrease in neuron survival; the cell borders were unclear and the Nissl pattern was blurred. The Nissl staining showed that FGF15-GNLs-treated animals recovered and the number of the spinal cord anterior horn neurons was increased. The FGF15-GNLs group had better spinal cord function recovery and treatment effects compared with those in the FGF15 group (Fig. 4A, B).

### FGF15-GNLs inhibits autophagy-induced cell death in SCI model

**Fig. 5:**
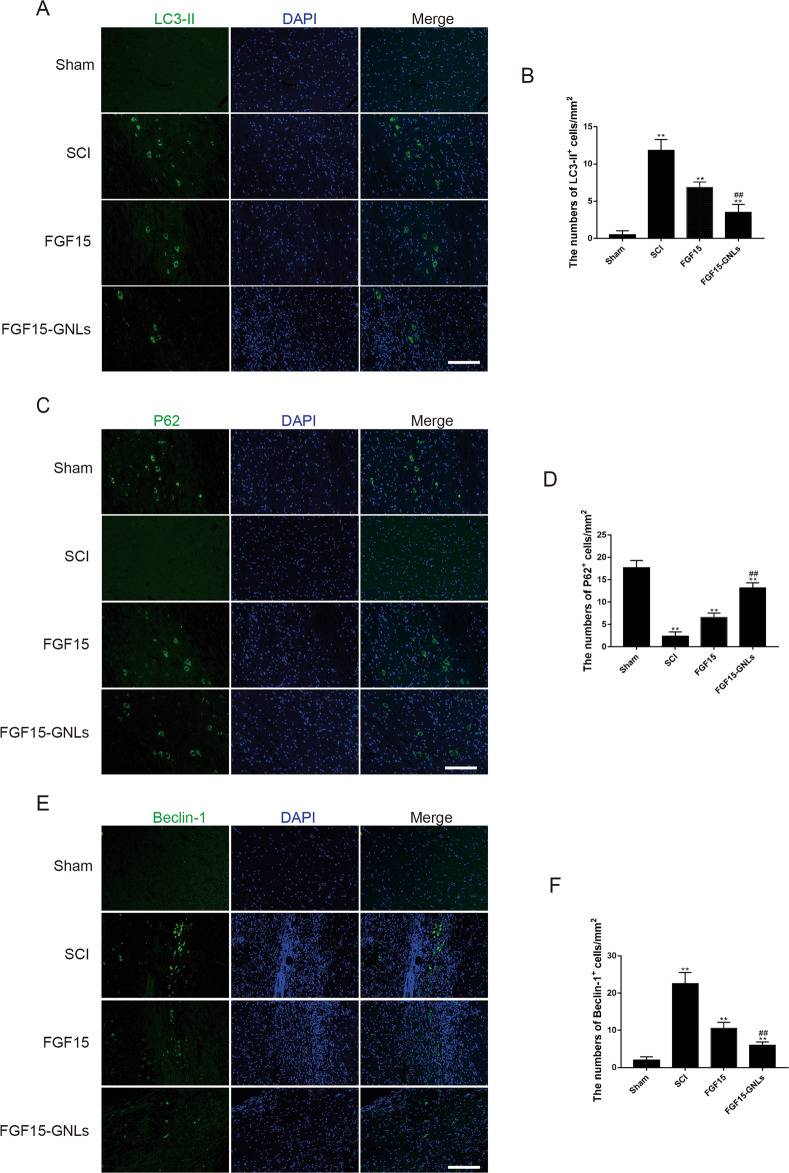
FGF15-GNLs inhibits autophagic cell death in SCI model. **A**, **C**, **E** The immunofluorescence staining for LC3-II, P62 and Beclin-1 immunostaining in the longitudinal spinal cord section from an animal in sham, SCI group, FGF15 group and FGF15-GNLs group, illustrating lesion morphology at the day of 60 after SCI. Green fluorescence represents LC3-II, P62 and Beclin-1. The nuclear is labelled by DAPI (blue). Scale bar = 100 μm. **B**, **D**, **F** Analysis of the positive cells and optical density of the Immunofluorescence staining results. **P < 0.01 vs. the sham or SCI group, ^##^*P* < 0.01 vs. the FGF15 group. Data are the mean values ± SEM, *n* = 6.

To determine whether the mechanism of FGF15-GNLs is related to the regulation of autophagy, Immunofluorescence staining for LC3-II was performed (Fig. 5A). The number of LC3-II positive cells was decreased in the FGF15-GNLs and FGF15 groups compared with that in the SCI group. The FGF15-GNLs group had less LC3-II-positive cells than that in the FGF15 group (*P* < 0.01) (Fig. 5B). p62 is one of the marker proteins that reflects the activity of autophagy and p62 content indirectly reflects the level of autophagy clearance. The number of p62-positive cells was increased in the FGF15-GNLs and FGF15 groups compared to that in the SCI group. The FGF15-GNLs group had more LC3-II-positive cells than that in the FGF15 group (*P* < 0.01) (Fig. 5C, D). Beclin-1 is required for the initiation of the formation of the autophagasome in autophagy. The number of beclin-1-positive cells was decreased in the FGF15-GNLs and FGF15 groups compared with that in the SCI group. The FGF15-GNLs group had less beclin-1-positive cells than that in the FGF15 group (*P* < 0.01) (Fig. 5E, F). The data indicate that FGF15 can inhibit autophagy and improve autophagy clearance thus inhibiting autophagy. The FGF15 group had better inhibition than that in the FGF15 group.

### FGF15-GNLs inhibits glial scar formation, promotes axon regeneration and expansion over the scar boundary and stimulates nerve regeneration

**Fig. 6:**
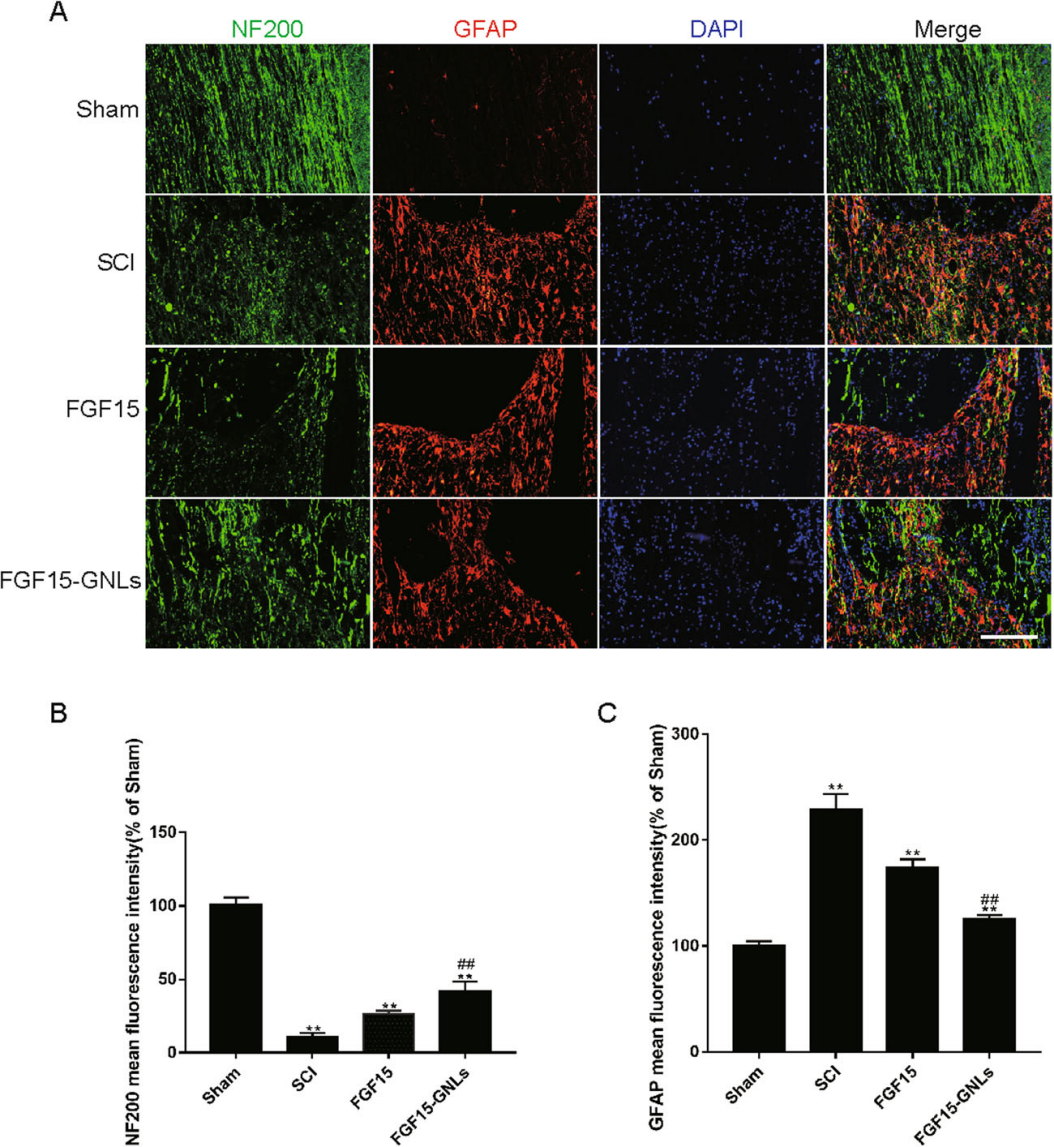
FGF15-GNLs inhibition of glial scar formation, promotion of axon regeneration expanding over the scar boundary and nerve regeneration. **A** Immunofluorescence staining results of NF200 and GFAP in the sham, SCI group, FGF15 group and FGF15-GNLs group. The bright green dots are considered as NF200-positive staining nerve regeneration. The bright red dots are considered as GFAP-positive staining glial scarring. The nuclear is labelled by DAPI (blue), scale bar = 100 μm. **B** Analysis of the NF200-positive cells reflect intensity of fluorescence results. **C** Analysis of the GFAP-positive cells reflect intensity of fluorescence results. **P < 0.01 vs. the sham group or SCI group, ^##^*P* < 0.01 vs. the FGF15 group. Data are the mean values ± SEM, *n* = 6.

Confocal analysis showed that NF200 expression in the FGF15-GNLs group was the highest compared to that in other treatment groups on day 60 (Fig. 6A). Interestingly, NF200 expression was present as a tree-branching contour in the FGF15-GNLs group most likely resembling that of the sham operation group, which is indicative of axonal sprouting ex vivo (*P* < 0.01) (Fig. 6B).

SCI predominantly results in glial scarring, which is generally formed by astrocytes interacting with the fibrous tissue partially dependent on upregulation of GFAP^26,27^. GFAP expression was the lowest in the FGF15-GNLs group on day 60 (Fig. 6A), indicating inhibition of glial scar formation in the FGF15-GNLs group. Confocal analysis showed that GFAP-expressing cells were restricted to the injury site within the scar line. The expression levels of GFAP in the FGF15-GNLs and FGF15 groups were significantly lower than that in the SCI group, and the expression levels of GFAP in the FGF15-GNLs group were lower than that in the FGF15 group (*P* < 0.01) (Fig. 6C). Overall, our data indicate maximal improved efficacy in astrocyte scar inhibition, axon regeneration expanding over the scar boundary and enhanced of axon regeneration in the FGF15-GNLs group.

## Discussion

FGF15 is a member of the family of neurotrophic factors and has broad mitogenic and cell survival activities; in addition, FGF15 is involved in various biological processes, including embryonic development, cell growth, morphogenesis, tissue repair and tumour growth and invasion^23,28,29^.

In the peripheral nervous system, FGF15 acts to promote the development of neurons to enhance their survival and maturation; in the central nervous system, FGF15 protects mature neurons^30^. The functional healing of adult rats with hemisected spinal cord was associated with significantly upregulated expression of endogenous FGF15 associated with neurogenesis and neuroprotection.

The application of the stimulators in SCI is aimed to have neuroprotective effects. However, most injected drugs are usually eliminated before any additional enhancements are manifested thus failing to meet the expectation. Therefore, it is important to develop the methods for induction of neurogenesis and neuroprotection in a physiologically sustainable manner.

Thus, the protective effect of exogenous FGF15 was used to promote the recovery after SCI. The data indicate that FGF15 can improve neuronal survival by reducing necrosis and nuclear pyknosis, which are considered the key components of the effective regeneration of the nervous system.

In this context, liposomes are an alternative means for transport and distribution of proteins. GNLs are microspherical particles produced by cholesterol, nontoxic surfactants, sphingolipids, glycolipids, long-chain fatty acids and even membrane proteins^31^. Unlike other carriers, liposomes have advantageous biocompatibility, biodegradability, toxicity and immune safety. This molecular transport mechanism improves the efficiency and solubility of the drugs, reduces side effects and virulence, releases drugs directly in specific sites in a controlled manner, reduces toxicity, improves bioavailability and changes pharmacokinetics.

However, FGF15 has not been used in clinic due to the following reasons: limited shelf life and high cost of production. Moreover, FGF15 is unstable and vulnerable to environmental changes such as temperature and pH^32^. Gelatine nanolipid carriers are regarded as a new delivery tool introduced after solid nanolipid particles. The mixture of solid and liquid lipids is used to prepare these particular structures^33^. The particles are widely used to enhance protection and stability of various agents because of their unique advantages of low toxicity and biodegradability. Therefore, GNLs may be a successful drug delivery system for FGF15. Certain stable GNLs in ethanol solutions were shown to penetrate the bilayer structure and increase the drug flow. Encapsulation enhances intracellular penetration of the molecules, transfers proteins through the blood–brain barrier and lowers side effects of the drugs^34^. Bioavailability of FGF15 delivered locally to the skin can be boosted by encapsulation. The delivery systems must be able to protect FGF15 from the environmental conditions and can function for a long time by continuously releasing the active drug to the target site. Thus, FGF15-GNLs have been developed; the data indicate that the particles are a stable and safe agent that can be used for the treatment of SCI. SCI involves a variety of destructive processes that are caused by a variety of factors that interfere with ionic homoeostasis and cause local oedema, focal haemorrhage and an increase in free radicals and free fatty acids. Autophagy is initiated by these stimuli and can additionally result from signals present during cellular differentiation and embryogenesis and on the surface of damaged organelles^35^.

Autophagic death of neurons and glial cells was shown to play a role in SCI-induced neuronal damage, and inhibition of these two processes may be a therapeutic strategy in SCI^36,37^. The consequences of pathological changes in the neurons cause damage to the neurons and glial cells and ultimately impair mobility estimated by the BBB scale. Certain studies suggested that autophagy signalling may have a direct role in promoting cell death in neuronal injury. Long-term stress and progressive autophagy can eventually result in cell death. Stimulation of excessive autophagy and cellular self-consumption may exceed the damage threshold of the cell and result in cell death^38–41^. During autophagy, p62, one of the ubiquitin-binding proteins, ubiquitinates proteins^42^ and forms a complex located on the inner membrane of the autophagosome, which is degrades in autophagic lysosomes. Thus, once autophagy is initiated, p62 undergoes a series of degradations in the cytoplasm^43^; when autophagy activity is weakened and autophagy function is defective, p62 will continuously accumulate in the cytoplasm. Beclin-1 and LC3-II are important markers of the level of autophagy. The beclin-1 and LC3-II levels are significantly increased during autophagy^44^. At day 60 after SCI, rats with increased p62 and deficiency in beclin-1 and LC3-II signalling showed increased residual white matter and enhanced exercise recovery. Elevated levels of p62 in SCI rats indicate that p62-mediated autophagy may be involved in SCI.

In conclusion, our study shows that the treatment with FGF15-GNLs increases neuronal recovery during SCI and promotes the motor function recovery. Inhibition of autophagy-induced cell death and glial scar and an increase in the levels of the neuroprotective factor NF200 were closely associated with the role of FGF15-GNLs in reduction of neuronal death in vivo suggesting that FGF15-GNLs is one of the treatment options for protection against cell death caused by autophagy; FGF15-GNLs may have a considerable impact on the treatment of central nervous system injury.

## Data Availability

The data applied in support of the conclusions of this study are of access from the correspondingng author upon request.
