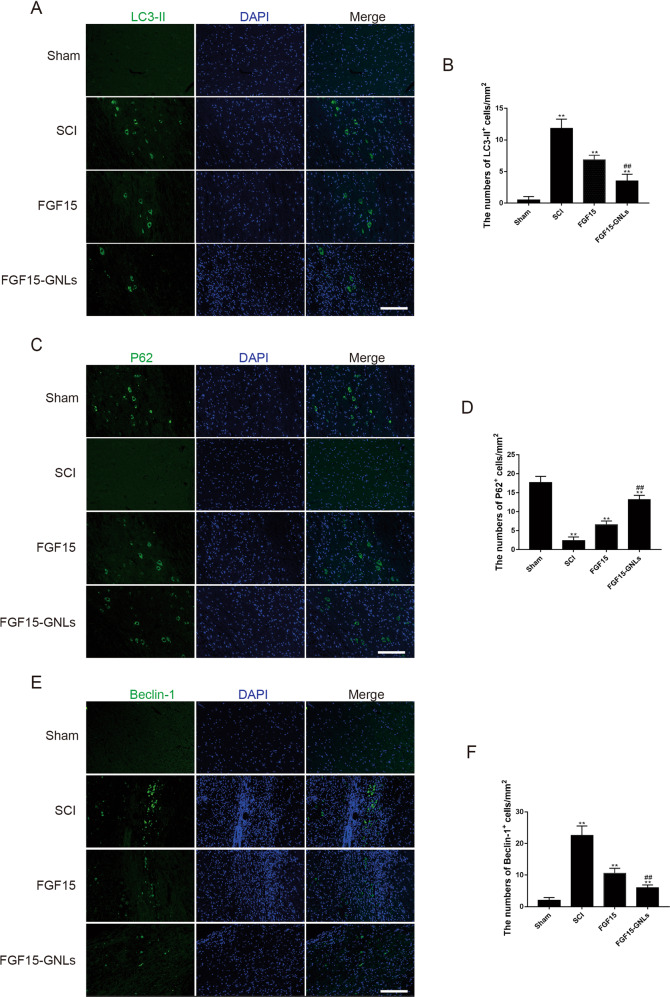# Author Correction: Gelatine nanostructured lipid carrier encapsulated FGF15 inhibits autophagy and improves recovery in spinal cord injury

**DOI:** 10.1038/s41420-022-00944-3

**Published:** 2022-04-04

**Authors:** Yibo Ying, Guangheng Xiang, Min Chen, Jiahui Ye, Qiuji Wu, Haicheng Dou, Sunren Sheng, Sipin Zhu

**Affiliations:** 1 Department of Orthopaedics, The Second Affiliated Hospital and Yuying Children’s Hospital of Wenzhou Medical University, Wenzhou, Zhejiang, 325000 China; 2 The Second School of Medicine, Wenzhou Medical University, Wenzhou, 325027 China

Correction to: *Cell Death Discovery* 10.1038/s41420-020-00367-y, published online 02 December 2020

The original version of this article unfortunately contained a mistake in figure 5. The correct figure can be found below. The authors apologize for the error. The original article has been corrected.